# Outcome at 1 year in patients with femoral shaft fractures treated with intramedullary nailing or skeletal traction in a low-income country: a prospective observational study of 187 patients in Malawi

**DOI:** 10.1080/17453674.2020.1794430

**Published:** 2020-07-23

**Authors:** Linda Chokotho, Hao-Hua Wu, David Shearer, Brian C Lau, Nyengo Mkandawire, Jan-Erik Gjertsen, Geir Hallan, Sven Young

**Affiliations:** a Department of Surgery, College of Medicine, University of Malawi; b Department of Clinical Medicine, University of Bergen, Bergen, Norway; c Institute for Global Orthopedics and Traumatology, Orthopedic Trauma Institute, University of California San Francisco, San Francisco, CA, USA; d Department of Orthopedic Surgery, Duke University Medical Centre, Durham, NC, USA; e Department of Orthopedic Surgery, Haukeland University Hospital, Bergen, Norway; f School of Medicine, Flinders University, Adelaide, Australia; g Department of Surgery, Kamuzu Central Hospital, Lilongwe, Malawi

## Abstract

Background and purpose — Intramedullary nailing (IMN) is underutilized in low-income countries (LICs) where skeletal traction (ST) remains the standard of care for femoral shaft fractures. This prospective study compared patient-reported quality of life and functional status after femoral shaft fractures treated with IMN or ST in Malawi.

Patients and methods — Adult patients with femoral shaft fractures managed by IMN or ST were enrolled prospectively from 6 hospitals. Quality of life and functional status were assessed using EQ-5D-3L, and the Short Musculoskeletal Function Assessment (SMFA) respectively. Patients were followed up at 6 weeks, 3, 6, and 12 months post-injury.

Results — Of 248 patients enrolled (85 IMN, 163 ST), 187 (75%) completed 1-year follow-up (55 IMN, 132 ST). 1 of 55 IMN cases had nonunion compared with 40 of 132 ST cases that failed treatment and converted to IMN (p < 0.001). Quality of life and SMFA Functional Index Scores were better for IMN than ST at 6 weeks, 3 and 6 months, but not at 1 year. At 6 months, 24 of 51 patients in the ST group had returned to work, compared with 26 of 37 in the IMN group (p = 0.02).

Interpretation — Treatment with IMN improved early quality of life and function and allowed patients to return to work earlier compared with treatment with ST. Approximately one-third of patients treated with ST failed treatment and were converted to IMN.

The gold standard treatment for femoral shaft fractures is intramedullary nailing (IMN), with low complication rates ranging from 1.2% to 5% for postoperative infection (Brumback et al. 2006, Young et al. 2013a, Salawu et al. 2017) and high union rates ranging from 72% to 100% (Ricci et al. 2001, El Moumni et al. 2009, Young et al. 2013b). However, nonoperative treatment using skeletal traction (ST) for at least 6 weeks remains the mainstay treatment for these fractures in low-resource settings (Hollis et al. 2015, Kramer et al. 2016). Nonoperative treatment is associated with increased risk of both medical and surgical complications, reported as high as 55% in some studies (Bucholz and Jones 1991, Doorgakant and Mkandawire 2012, Kramer et al. 2016, Parkes et al. 2017).

In Malawi, femoral shaft fractures are most commonly treated by ST. IMN, when performed, is done using the SIGN IM nail, which is donated by SIGN Fracture Care International (Richland, WA, USA) (Shah et al. 2004). Most studies comparing IMN with ST in LICs used conventional measures such as fracture union, complications, and range of motion (Swai 2005, Kamau et al. 2014, Parkes et al. 2017). No prior study has measured quality of life or function using a validated patient-reported outcome instrument to compare ST and IMN in any context.

This study compared the quality of life and functional status of patients with femoral shaft fractures treated with either ST or IMN in Malawi.

## Patients and methods

### Study setting and patient enrolment

**Figure 1.**
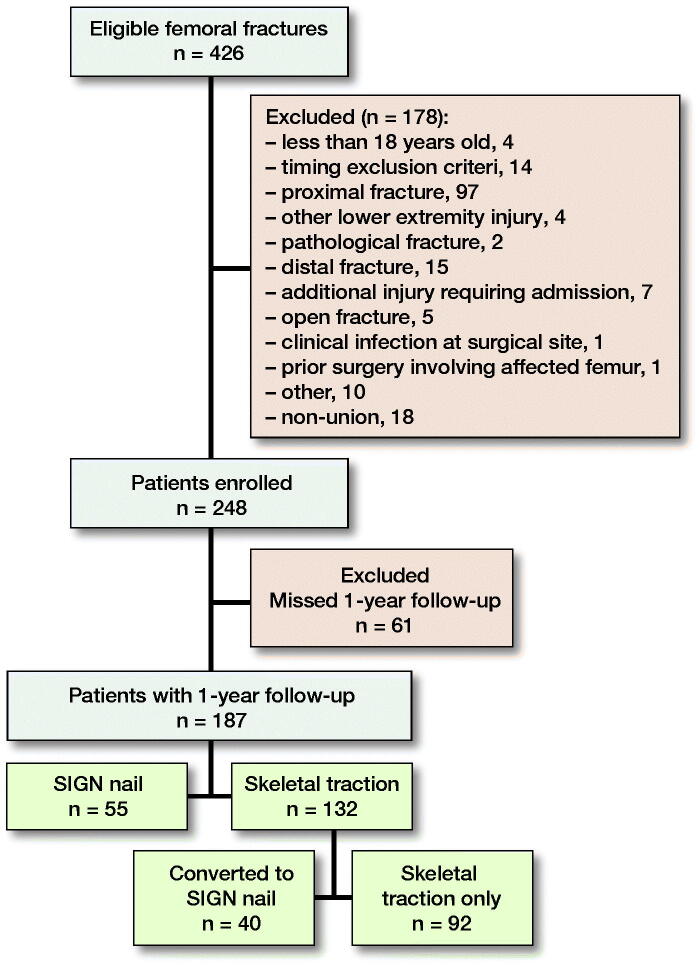
Flow chart showing eligibility, exclusion, enrolment and loss to follow-up of patients.

This is a prospective multicenter observational study where adult patients aged 18 years and older, with isolated unilateral femoral shaft fractures (AO/OTA class 32) in 6 hospitals in Malawi, were enrolled from March 2016 to July 2018. Patients with associated major injuries, pathological or open fractures, infection at the surgical site, or prior surgery involving the affected femur were excluded (Figure 1).

The type of treatment (ST or IMN) was determined by the treating orthopedic clinical officer (OCOs) or surgeon. OCOs are non-physician clinicians trained to provide nonoperative care for orthopedic conditions and emergency orthopedic surgery for selected cases, such as acute infections and open fractures (Mkandawire et al. 2008).

The patients were recruited from Queen Elizabeth Central Hospital (QECH), Kamuzu Central Hospital (KCH), Beit Cure International Hospital (BCIH), and 3 district hospitals: Chiradzulu, Thyolo, and Chikwawa. In both QECH and KCH, patients with femoral shaft fractures were treated with ST or IMN based on the treating clinician’s assessment, which was based largely on surgical capacity of the hospital at that time. In the district hospitals all patients were treated by ST. IMN patients who met the inclusion criteria were recruited into the study if they had surgery within 6 weeks from the time of injury. ST patients either continued with skeletal traction until clinical and radiological signs of fracture union were present or were offered IMN if, in the opinion of the treating clinician, union was unlikely without further intervention. The diagnosis of delayed union was made by the treating clinician, if at 6 weeks or more post-injury there was still tenderness and mobility at the fracture site, and no radiological evidence of callus formation. Nonunion was defined as no evidence of fracture healing both clinically and radiologically after at least 3 months on ST or 6 months after IMN. Consequently, the ST group had 2 sub-groups: those who started with skeletal traction but later converted to IMN because of either delayed union or nonunion and those who had skeletal traction as definitive treatment until union. A sample size of 110 patients in each group was initially calculated using OpenEpi software (www.openepi.com) (Sullivan et al. 2009) at 95% confidence interval and 80% power using the minimal clinically important difference (MCID) (Jaeschke et al. 1989) of 0.1 between the 2 groups for the EQ-5D, with a standard deviation of 0.12 (Luo et al. 2010, Ibrahim et al. 2018) and a more conservative standard deviation of 0.2 was used for the ST group. The calculation was adjusted to account for 20% loss to follow-up. However, at the 1-year interim analysis there were 65 patients in the IMN group and 120 patients in the ST group. A new sample size was calculated with an allocation ratio of 2:1, resulting in a required sample size of 80 cases in the IMN group and 160 patients in the ST group.

### Treatment

The SIGN nail was used in all IMN patients. This is a solid locking IM nail that can be inserted without need for a fracture table or intraoperative fluoroscopy. At KCH and QECH, the SIGN nail was inserted using open reduction on a standard operating table. At BCIH, fluoroscopy guidance was used.

All ST patients had straight leg extension skeletal traction with a Steinmann pin inserted into the proximal tibia under local anesthesia, using an aseptic technique. A stirrup, rope and weights assembly was hung over a bar, pulley, or directly over the end of the bed. Counter-traction and anti-rotating mechanisms were used at the treating clinician’s discretion. Pin site care was performed daily by the patients’ guardians.

All patients received physiotherapy by either the hospitals’ physiotherapists or rehabilitation technician.

### Outcomes

The primary outcomes were quality of life determined by European Quality of Life 5-Dimensions Survey (EQ-5D-3L) index score (Brooks and Group 1996) and the Short Musculoskeletal Functional Assessment (SMFA) Function and Bothersome index scores (Swiontkowski et al. 2005). Both tools have been translated to Chichewa and validated in Malawi (Chokotho et al. 2017, 2019). Both tools were administered verbally by the research assistants who recorded the responses on Microsoft surface computers.

Index utility scores for the EQ-5D-3L were generated using the value set for the Zimbabwean population (Jelsma et al. 2003).

At each follow-up, patients were asked if they had returned to their pre-injury work, whether employed or otherwise. No specification was made as to whether the patients did not return to work because of the injury or because they were laid off due to injury-related absenteeism.

### Follow-up

Follow-up assessments were performed 6 weeks, 3 months, 6 months, and 1 year after injury. If patients missed scheduled appointments, a telephone interview to answer the EQ-5D-3L and SMFA questionnaires was undertaken.

Patients who failed to come for an appointment and were not reached by phone were assessed by research assistants in their homes. Patients who could not be contacted by telephone and could not be found in person were regarded as lost to follow-up.

### Statistics

Data were collected using RedCap electronic data capture tools hosted at the University of California San Francisco (UCSF) (Harris et al. 2009). Data were analyzed using Stata version 10.0 (StataCorp, College Station, TX, USA). Unadjusted analysis was done between IMN and ST groups using Satterthwaite’s t-test for means with unequal variances. Sub-group analysis was also done between the IMN group and successful ST patients. Potential confounders associated (not necessarily causally related) with the outcome were first identified in a univariate regression analysis. Marital status, mechanism of injury, and education level were identified as significantly associated with both the EQ-5D and SMFA scores. The potential confounders and other independent variables were then added in a generalized linear regression model using the forward stepwise regression approach to come up with a final model. Comparison of categorical data was done using a chi-square test, or Fisher’s exact test when any expected cell frequency was less than 5. Listwise deletion of missing data was used in unadjusted and adjusted regression analysis. Findings were considered statistically significant if the p-value was less than 0.05, thus “significant” results refers to statistical significance. Clinical significance is presented using MCID. Estimates were presented with their 95% confidence intervals (CI).

### Ethics, funding, and potential conflicts of interest

The study was approved by the College of Medicine Research Ethics Committee, in Malawi, and the University of Bergen and University of California San Francisco Institutional Review Boards. Written informed consent was obtained from all patients in the study. The study was funded by the Institute of Global Orthopedics and Traumatology (IGOT), University of California San Francisco, James O. Johnston Research Grant, and a PhD grant through the Norhed Project, financed by Norad. Author DS is a non-paid member of the Board of Directors for SIGN Fracture Care International. The rest of the authors declare no conflict of interest.

## Results

There were 426 eligible cases, of which 248 were enrolled in the study. 1-year follow up was achieved in 187 cases (75%) (Figure 1). 55 and 132 cases were treated with IMN and ST respectively.

### Baseline demographic and injury details

The mean age of patients was 38 (SD 13) years in the IMN group and 40 (SD 16) years in the ST group (Table 1). In both groups the majority of patients were male. The most common cause of injury was road traffic injury followed by falls. More people in the ST group had primary school as their highest level of education, whereas there were more people with post-secondary education in the IMN group (p < 0.001). Most fractures were AO/OTA type 32A, but there were more type 32B in the IMN group than in the ST group, p = 0.02 (Table 1).

**Table 1.** Baseline details

Variable	IM nailing n = 55	All skeletal traction n = 132	p	Convert n = 40	Successful traction only n = 92
Age, mean (SD)	38 (13)	40 (16)	0.3	37 (14)	41 (17)
median	37	37 (			
IQR	28–45	26–48			
Sex, n (%)			0.7		
Female	12	22 (17)		6	16
Male	42	107 (81)		33	74
Missing	1	3 (2)		1	2
Marital status			0.8		
Single	16	39 (30)		10	29
Married	36	79 (60)		26	53
Divorced/separated	1	5 (3.8)		2	3
Widow/widower	2	7 (5)		1	6
Missing	0	2 (2)		1	1
Education			< 0.001		
Primary	13	76 (58)		16	60
Secondary	18	40 (30)		18	22
Post-secondary	22	12 (9)		5	7
Missing	0	4 (3)		1	3
Mechanism of injury			0.4		
Fall	13	45 (34)		12	33
RTI	37	68 (52)		24	44
Other	4	16 (12)		2	14
Missing	1	3 (2)		2	1
Smoking			0.3		
No	52	112 (85)		33	79
Yes	2	13 (10)		4	9
Missing	1	7 (5)		3	4
OTA classification			0.02		
A (simple)	37	97 (74)		31	66
B (wedge)	13	15 (11)		5	10
C (complex)	4	5 (4)		1	4
Missing	1	15 (11)		3	12
OTA 32A subclass			0.06		
Oblique	10	11 (8)		4	7
Spiral	6	16 (12)		7	9
Transverse	18	67 (51)		20	47
Missing	21	38 (29)		9	29
Location			0.4		
Distal zone	3	16 (12)		6	10
Middle zone	35	82 (62)		27	55
Subtrochanteric	9	14 (11)		3	11
Missing	8	20 (15)		4	16
Side of injury			0.7		
Right	29	70 (53)		22	48
Left	23	53 (40)		14	39
Missing	3	9 (7)		4	5
Duration before treatment		< 0.001			
mean (SD)	13 (12)	4.4 (5)		6 (6)	5 (12)
median	10	3		3	3
IQR	3–18	1–6		1–8	1–5

### Treatment

The mean waiting time from injury to definitive treatment was 13 (SD 12) days for the IMN group and 4.4 (SD 5) days for the ST group, p < 0.001 (Table 1). 1 patient in the IMN group had a nonunion and was treated with an exchange nail, whereas 40 patients (30%) in the ST group had either nonunion or delayed union and subsequently converted to IMN during the course of the study (p < 0.001). Details on duration from time of injury to conversion were available for 20 patients out of 40, with a median of 63 days and a range of 50 to 252 days.

### Quality of life

#### IMN versus all ST patients

**Figure 2.**
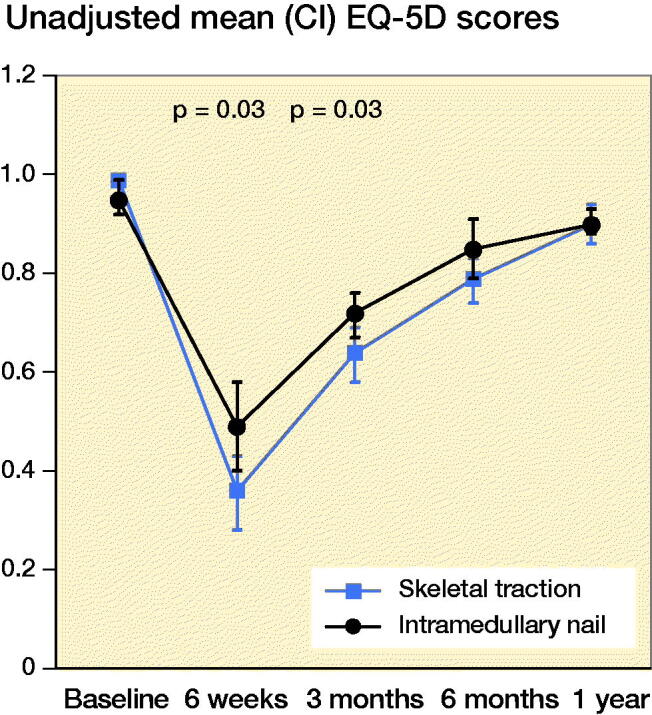
Unadjusted mean EQ-5D scores for IM nailing vs. skeletal traction.

The unadjusted mean EQ-5D index scores were higher in the IMN group than ST group at 6 weeks (p = 0.03) and 3 months (p = 0.03) after injury (Figure 2) but not at 6 months and 1 year. The mean EQ-5D index scores were lower at 1-year post injury compared with baseline, (p < 0.001). Patients in the IMN group reported significantly better quality of life than those in the ST group at 6 weeks, 3 months, and 6 months after the injury, with an adjusted mean difference of –0.14 (CI –0.27 to –0.02); –0.07 (CI –0.14 to –0.0001); –0.08 (CI –0.15 to –0.01) respectively. The mean difference was greater than MCID at 6 weeks and equal to MCID at 3 months and 6 months (Table 3).

#### Successful skeletal traction versus IMN

There were no significant differences in the unadjusted and adjusted mean EQ-5D index scores between patients who were treated successfully with ST (without converting to IMN) and those patients who were treated primarily with IMN (Tables 2 and 3). However, the adjusted mean difference in index scores was similar to MCID at the 6 weeks (–0.09, CI –0.2 to 0.06) and 3 months intervals (–0.07, CI –0.2 to 0.03) (Table 3).

**Table 2.** Unadjusted results for sub-group analysis. Values are mean (CI)

Variable	Pre-injury/baseline	6 weeks	3 months	6 months	1 year
EQ-5D					
Successful ST	0.99 (0.98–1)	0.40 (0.31–0.49)	0.64 (0.50–0.73)	0.80 (0.74–0.86)	0.91 (0.88–0.93)
IMN	0.95 (0.92–0.99)	0.50 (0.42–0.59)	0.72 (0.68–0.77)	0.85 (0.78–0.91)	0.91 (0.87–0.95)
SMFA FI					
Successful ST	1.5 (1.0–2.0)	52 (48–57) **^a^**	36 (29–42) **^a^**	23 (18–28) **^a^**	6.7 (4.9– 8.5)
IMN	2.5 (0.8–4.1)	43 (38–47)	27 (23–31)	16 (11–20)	9.3 (5.7–13)
SMFA BI					
Successful ST	0 (	48 (43–54) **^a^**	30 (24–37)	18 (13–23)	6.3 (4.1–8.4)
IMN	1 (–0.4 to 2)	39 (34–44)	24 (19–29)	13 (7.9–18)	7.6 (3.6–12)

**^a^** statistically significant (p < 0.05)

SMFA FI, SMFA Function Index. SMFA BI, SMFA Bothersome Index

**Table 3.** Adjusted results

Variable	Pre-injury/baseline coefficient (CI)	6 weeks coefficient (CI)	3 months coefficient (CI)	6 months coefficient (CI)	1 year coefficient (CI)
ST vs. IMN					
EQ5D score	0.03 (–0.004 to 0.1)	–0.14 (–0.27 to –0.02)	–0.07 (–0.14 to –0.0001)	–0.08 (–0.15 to –0.01)	0.001 (–0.05 to 0.05)
p-value	(0.1	(0.03	(0.05	(0.04	(1
SMFA FI	–1.0 (–2.5 to 0.6)	8.7 (2.6 to 15)	8.4 (2.6 to 14)	7.9 (1.7 to 14)	–2. (–5.8 to 1.7)
p-value	(0.2	(0.01	(0.01	(0.01	(0.3
SMFA BI	–0.5 (–1.9 to 0.9)	9.2 (2.4 to 16)	7.7 (1.2 to 14)	6.7 (–0.3 to 14)	–1.2 (–5.4 to 2.9)
p-value	(0.5	(0.01	(0.02	(0.1	(0.6
IMN vs. successful ST					
EQ5D score	0.03 (–0.002 to 0.1)	–0.09 (–0.2 to 0.06)	–0.07(–0.2 to 0.03)	–0.05 (–0.14 to 0.03)	–0.0001 (–0.05 to 0.05)
p-value	(0.1	(0.2	(0.2	(0.2	(1
SMFA FI	–1.1 (–2.6 to 0.5)	8.5 (1.8 to 15)	7.6 (0.4 to 15)	7.2 (0.4 to 14)	–2.4 (–6.3 to 1.5)
p-value	(0.2	(0.01	(0.04	(0.04	(0.2
SMFA BI	–0.9 (–2.2 to 0.4)	8.8 (0.9 to 17)	5.5 (–2.5 to 14)	4.1(–3.6 to 12)	–1.2 (–5.6 to 3.1)
p-value	(0.2	(0.03	(0.2	(0.3	(0.6

### Functional status

#### IMN versus all ST patients

**Figure 3.**
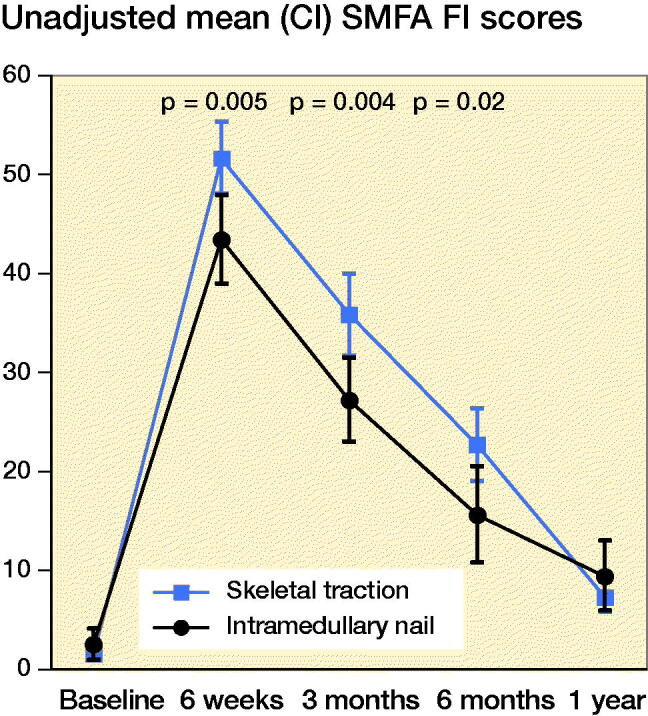
Unadjusted mean SMFA Functional Index scores for IM nailing vs. skeletal traction.

Both unadjusted and adjusted analyses showed significantly lower mean SMFA functional index scores at 6 weeks, and 3 and 6 months post-injury in the IMN group, indicating better function compared with the ST group (Figure 3 and Table 3).

**Figure 4.**
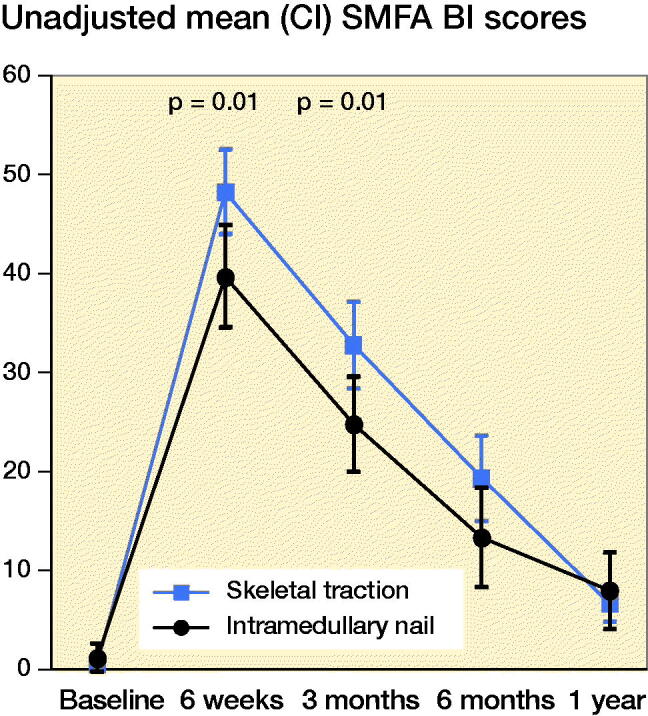
Unadjusted mean SMFA Bothersome Index for IM nailing vs. skeletal traction.

Further, the unadjusted mean SMFA Bothersome index was significantly lower in the IMN group compared with the ST group at 6 weeks and 3 months post-injury, indicating that patients in the IMN group were less bothered by their condition (Figure 4). Adjusted analysis showed a similar trend with mean difference in the SMFA Bothersome index of 9.2 (CI 2.4–16) at 6 weeks and 7.7 (CI 1.2–14) at 3 months (Table 3).

### Successful skeletal traction vs. IMN

The mean SMFA functional index scores were significantly lower in the IMN group compared to the successful ST group at 6 weeks, and 3 and 6 months post-injury for both unadjusted (Table 2) and adjusted analysis (8.5, CI 1.8–15; 7.6, CI 0.4–15; 7.2, CI 0.4–14), (Table 3).

The unadjusted and adjusted mean SMFA Bothersome index scores were significantly lower in the IMN group compared with the successful ST group at 6 weeks (Tables 2 and 3).

### Return to work

88 of 103 cases followed up at 6 months responded to the question of whether they had returned to work. No reasons were specified for non-response to this question in the remaining 15 cases (9 in the IM group and 6 in the ST group). 24 of 51cases in the ST group had returned to work compared with 26 of 37 in the IMN group (p = 0.02). There were no significant differences in proportions of patients who had returned to work at the other follow-up time points.

## Discussion

This study found improved quality of life and function up to 6 months post-injury for IMN compared with ST in patients treated for femoral shaft fractures in Malawi. Almost one-third of patients treated with ST failed treatment and were ultimately converted to IMN due to delayed union or nonunion, typically between 6 and 12 weeks after initiating traction. Nonetheless, patients achieving union with skeletal traction had equivalent outcomes to those treated with early IMN at 1 year.

As far as we know, this is the first study comparing quality of life and functional status in femoral shaft fracture patients treated with ST or IMN. Haug et al. (2017) looked at quality of life in femoral shaft fracture patients treated with skeletal traction and found that patients had both physical and psychological pain as well as emotional distress due to prolonged hospitalization and the associated negative economic impact on their families. Tay et al. (2014) found that patients with long bone diaphyseal fractures treated surgically still had residual physical impairment and pain in the first year post-injury, which was worse among those with delayed union and nonunion even after treatment. Ibrahim et al. (2018) also found that EQ-5D scores did not return to the pre-injury level after operative treatment of femoral shaft fractures, a finding that was also replicated in our study. These studies support the concept that long bone fractures affect long-term quality of life and functional status even after operative treatment.

Patients treated with skeletal traction are normally admitted to hospital for at least 6 weeks, which is likely to have substantial financial implications for the patients, their guardians, and the health service providers. In our study, less than half of the ST patients had returned to work at 6 months after the injury compared with approximately three-quarters in the IMN group. The direct and indirect costs associated with skeletal traction may be more than the cost of intramedullary nailing. A cost-effectiveness study of the 2 treatment modalities is needed to give a complete picture of the impact of the treatment modalities and the findings could assist in better priority setting and resource allocation.

One-third of the ST patients were converted to IMN due to either delayed union or nonunion. These findings highlight the unmet need for operative fracture treatment in Malawi, where patients are offered operative treatment mostly after failure of primary nonoperative treatment, despite clear evidence in the literature that operative treatment is superior (Brumback et al. 2006, Kamau et al. 2014, Chagomerana et al. 2017). Femoral shaft nonunion is incapacitating and its impact on health-related quality of life is comparable to severe hip osteoarthritis and worse than medical conditions such as myocardial infarction and congestive cardiac failure (Brinker et al. 2017). In addition, nonunion surgery is more complex than acute fracture surgery and has an increased risk of infection and other complications (Mahomed 2008, Young et al. 2013b), and also has the potential to use more resources. Efforts should therefore be made to improve surgical services and avert the problem of converting to IMN after failed skeletal traction.

Conducting clinical research in low-resource settings presents many challenges, and our study has several limitations. First, the IMN group was not homogeneous. The delay from time of injury to treatment ranged from 1 day to 6 weeks, signifying the challenges faced by orthopedic surgeons in Malawi to provide operative fracture care in a setting where theatre time is limited, and the few available specialists are overwhelmed by the large burden of fractures needing surgery. This baseline discrepancy may have contributed to suboptimal quality of life and function in the IMN group, as early operative stabilization of these fractures is associated with fewer complications and better outcomes in the short term (Mahomed 2008, El-Menyar et al. 2018). Lack of homogeneity also limits its external validity. Another limitation is that there was a high rate of conversion from ST to IMN due to either delayed union or nonunion. This occurred after at least 6 weeks on skeletal traction, and as a result there was no bias at 6 weeks. However, the remaining time points were likely biased towards the null hypothesis of no difference between groups because those patients who failed traction would have experienced a poor outcome had they continued with ST for the entire follow-up period. Details on post-treatment physiotherapy, which plays a crucial role in improving function after injury (Paterno and Archdeacon 2009), were not collected. However, patients in both groups were provided with standard rehabilitation by either the hospitals’ physiotherapists or rehabilitation technicians. Thus it is unlikely that post-treatment rehabilitation affected the functional outcome in 1 group more than the other. We also did not collect detailed information on comorbidities. However, as the mean age in both groups was less than 40 years it is unlikely that there were patients with substantial comorbidities. Loss to follow-up at the different time intervals may have reduced the power of the study to detect a statistically significant difference. Nonetheless, the differences were significant at early time points, and the mean difference found at 1 year was far below the MCID for the EQ-5D. Loss to follow-up also causes uncertainty with regard to the true effect of the treatment modalities, due to unknown outcomes of those who missed follow-up. However, Young et al. (2013b) found that the majority of the femoral shaft fracture patients in Malawi who did not return to hospital for follow-up were doing well. Another limitation was that there was no standard definition of delayed union and nonunion in the study’s facilities. As most patients routinely have only one radiographic view, either anteroposterior (AP) or lateral, it was not possible to use standard scoring systems such as the RUST Score (Whelan et al. 2010) or the criteria used by Tsang et al. (2016). Finally, because patients’ assignment to the 2 study groups was not randomized, there is a potential for confounding due to unmeasured baseline characteristics. Further, regression models may not adequately control for confounding (Shrier and Platt 2008). However, since only confounders measured at baseline were included, we argue that none of these can be colliders in the analysis. Nevertheless, this prospective observational study is the first to compare the quality of life and functional status of femoral shaft fractures treated with either an intramedullary nail or skeletal traction in a LIC.

In conclusion, this study found that treatment with IMN improved early (≤ 6 months) postoperative quality of life and function and allowed patients to return to work earlier compared with those treated with ST. Treatment of femoral shaft fractures with ST in a resource-limited setting may achieve similar outcomes to IMN in quality of life and function at 1-year post-injury if fracture union is achieved. However, approximately 1 in every 3 patients treated with straight-leg ST failed treatment, requiring conversion to surgical treatment. There is a need for a cost-effectiveness study comparing these 2 treatment modalities to gain a broader picture of the impact of treatment for femoral shaft fractures in low-resource settings.

LC designed the concept of study, supervised and monitored data collection, analyzed the data, and drafted the manuscript. BL provided input on the concept of the study, supervised and monitored data collection, and helped with database design and critical revision of the manuscript. HHW set up the database, and supervised and monitored data collection, and critical revision of the manuscript. DS provided input on the concept of the study, monitored data collection, and undertook critical revision of the manuscript. NM, JEG, GH, and SY provided input on the concept of the study, and undertook critical revision of the manuscript.

The authors would like to thank all the orthopedic surgeons and orthopedic clinical officers for their support during the study. They would also like to thank the study’s physiotherapists who did the clinical assessments. Special gratitude is due to Mr Foster Mbomuwa, the project’s coordinator, for all his efforts during the study.
